# Anxiety, trauma and well-being in health-care professionals during COVID-19 first wave in Spain: the moderating role of personal protection equipment availability

**DOI:** 10.1186/s12955-021-01845-2

**Published:** 2021-08-28

**Authors:** Miriam Bajo, Pilar Gallego, Maria Stavraki, Grigorios Lamprinakos, Pablo Luna, Darío Díaz

**Affiliations:** 1 Department of Medical Psychology, Ciudad Real Medical School, Universidad de Castilla La Mancha, Camino de Moledores S/N, 13071 Ciudad Real, Spain; 2 Department of Psychology, Ciudad Real Faculty of Education, Universidad de Castilla La Mancha, Ronda de Calatrava, 3, 13071 Ciudad Real, Spain; 3 Birmingham Business School, Edgbaston, Birmingham, B15 2TT UK

**Keywords:** Health-care professionals, Well-being, Complete state model of health, Anxiety

## Abstract

**Background:**

The effect of COVID-19 on Health-Care Professionals’ mental health has received increased attention in the last year’s literature. However, previous studies essentially evaluated psychopathological symptoms and not the presence of positive mental health. Therefore, the first objective of the present research is to evaluate health-care professionals’ mental illness (i.e., anxiety and traumatic intensity) and positive mental health (i.e., well-being) using the Complete State Model of Health. Our second objective is to study the effect of Personal Protection Equipment availability on professionals’ mental health.

**Methods:**

Two-hundred and thirty-two health-care professionals working in Spain in the first line of COVID-19 patient care participated in the study. To measure anxiety, traumatic intensity and well-being participants completed the State Trait Anxiety Inventory, the Davidson Trauma Scale, and the Mental Health Continuum-Short Form. Pearson correlations were used to examine the relationships between all scales. In order to test the two continua model of mental health, we used parallel analysis and exploratory factor analysis. To analyze anxiety, traumatic intensity, and well-being differences between health-care professionals with and without Personal Protection Equipment availability we conducted different ANOVAS. To test our hypothesis regarding the moderating role of Personal Protection Equipment availability in the effect of mental illness on positive mental health, data were subjected to a hierarchical regression analysis.

**Results:**

As in previous studies, health-care professionals showed high levels of anxiety and traumatic intensity. They also presented low levels of well-being indicators. According to our hypothesis, results of parallel analysis and exploratory factorial analysis indicated that the measures of mental illness and positive mental health loaded on separate but correlated factors. Finally, Personal Protection Equipment availability moderated the effects of state anxiety and traumatic intensity on professionals’ well-being.

**Conclusions:**

Health-care professionals’ mental illness and positive mental health reflect distinct continua, rather than the extreme ends of a single spectrum. Therefore, it is essential to measure both psychopathology and the presence of positive health to comprehensively evaluate professionals’ mental health. Finally, our results indicated that Personal Protection Equipment availability is essential not only for professionals’ physical health, but also for their mental health.

## Background

The multidisciplinary study of the disease caused by the Coronavirus SARS-CoV-2 called COVID-19 (Coronavirus Disease 2019) has received great interest due to its impact throughout the world. The coronavirus disease spread across the planet rapidly during the beginning of 2020 (first wave) which caused the World Health Organization (WHO) to consider the existence of a pandemic on March 11, 2020. This pandemic has led to the total or partial confinement of most of the world's population.

The European Union was one of the most affected areas during the first wave, with 517,443 cases diagnosed, and 48,600 deaths, according to data provided by the European Center for Disease Prevention and Control (ECDC) on April 4, 2020 (when the present study was conducted). Within Europe, Spain was dramatically affected causing a serious overload to the Spanish health system. On March 14, 2020, the national state of emergency was declared in Spain, which led to population’s home confinement to stop the progression of the epidemic. Even so, diagnosed cases increased considerably during the week of March 28 to April 4, 2020. In fact, on April 4, Spain had 124,736 confirmed cases (34,219 recovered), 57,612 hospitalized patients, 6,532 Intensive Care Units (ICU) patients and 11,744 deaths according to official data provided by the Spanish Government. This large increase in the number of patients caused an emergency reorganization in hospitals, creating a climate of uncertainty for health-care professionals. Furthermore, there was a great work overload, especially in ICU, with a very high ratio of patients/professionals, and many professionals had to attend patients even without the required clinical training. During this period, healthcare professionals were living in a constant fear of COVID-19 disease exposure, a fact that was aggravated by the lack of Personal Protection Equipment (PPE).

The factors previously mentioned make the study of health-care professionals’ mental health during the COVID-19 pandemic extremely crucial. Research addressing this issue has slowly begun to appear (e.g., [28, 32] although most studies have been conducted with Chinese populations. For example, a recent review of the impact of COVID-19 on health-care professionals’ mental health found that one in four reported depression and anxiety, and one in three suffered insomnia [40]. Also, an important limitation of previous research is that it only focused on the study of mental illness (i.e., psychopathological symptoms) and not on health-care workers’ positive health. The only exception, to the best of our knowledge, is the work by Hennein and Lowe [22]. Although this work only measures pathology (i.e., major depression, generalized anxiety disorder, post-traumatic stress disorder, and alcohol use disorder), the authors included two open-ended questions asking respondents to recount their most upsetting and hopeful experiences during the COVID -19 pandemic and how it made them feel. Through a qualitative analysis of the answers to these questions, the authors draw some conclusions about health-care professionals’ well-being. Therefore, despite the WHO recommendations to consider health as a complete state of physical, mental and social well-being, and not only as the absence of disease [52], to our knowledge, there is no quantitative research on the influence of COVID-19 on health-care professionals’ well-being.

Although there are different models that consider health as a continuum between an optimal state of positive mental health and a state of complete illness (e.g., [1], one of the most widely used in psychological research is the Complete State Model of Health (CSMH) suggested by Corey Keyes [26]. As already mentioned, the definition of health proposed by the WHO suggests that health is a state not only characterized by the absence of disease, but also by the presence of “something positive” [44, 52]. Concretely, to specify the factors of this positive state, the CSMH defines mental health as a set of symptoms of hedonia (emotional well-being) and positive functioning (psychological well-being and social well-being). After reviewing previous literature on well-being, Keyes proposed a total of 14 positive mental health indicators: happiness, interest, satisfaction with life, autonomy, environmental mastery, personal growth, positive relations with others, purpose in life, self-acceptance, social acceptance, social actualization, social contribution, social coherence and social integration. The first three indicators belong to Emotional Well-Being (EWB), the next six to Psychological Well-Being (PWB), and the last five to Social Well-Being (SoWB). The CSMH suggests that positive mental health (i.e., well-being) and mental illness are two distinct but interrelated domains of mental health. Therefore, as proposed by the *two-continua model of mental health* [26], mental illness and positive mental health reflect distinct continua, rather than the extreme ends of a single spectrum.

Although the CSMH has been applied to different populations and disorders (e.g., [2, 33], to the best of our knowledge, up to date there is no study applying this model to health-care professionals. Therefore, the first objective of the present research is to evaluate health-care professionals’ mental health using instruments designed not only to measure psychopathology (i.e., anxiety and stress) but also to measure well-being. According to previous research (e.g., [3, 25], we expect that two dimensions, positive mental health—mental illness will emerge in our sample of health-care professionals. Our second objective is to study the effect of PPE availability on health-care professionals’ mental health. Based on previous literature that indicates the absence of PPE as an important source of anxiety (e.g., [45], we expect that health-care professionals with access to the recommended PPE will show lower levels of pathology (i.e., anxiety and traumatic intensity) and greater levels of positive mental health (i.e. well-being) than health-care professionals without access to this equipment. We also expect that the availability of PPE will moderate the effect of anxiety and traumatic intensity (mental illness) on health-care professionals’ well-being (positive mental health).

## Methods

### Participants

Two-hundred and thirty-two participants between 25 and 65 years old voluntarily participated in the study without compensation. Participants were 204 women (87.9%) and 28 men (12.1%) with a mean age of 38.02 years (*SD* = 14.07). Participants were health-care professionals (medical doctors: 38.8%, nurses: 61.2%) working in the first line of COVID-19 patient care in public or private hospitals in the territories most affected by the pandemic first wave in Spain. They were recruited via a letter of invitation explaining the project and the voluntary nature of participation. Participants were selected to meet the following criteria: (1) being health-care professionals; (2) being in the front line of Covid-19 patient care at the time of the study (having a direct contact with COVID-19 patients); (3) work in a public or private hospital in the Community of Madrid, Castilla la Mancha, Castilla-León and Catalonia.

### Procedure

Participants completed the study during the first wave of the COVID-19 pandemic between March 28 and April 4, 2020, the week with the greatest overload in the Spanish health system due to the high number of new daily infections detected. This study was part of a research project funded by the Spanish Ministry of Science, and was approved by the ethics committee of the “Universidad de Castilla - La Mancha” (UCLM) and the HGUCR (“Comité Ético de Investigación Clínica HGUCR-UCLM”). All health-care professionals were informed that all collected information was confidential and anonymous, and signed an informed consent. Participants were provided with three questionnaires, which were presented in one of two orders to account for possible effects due to the order of presentation. Half the participants completed a booklet containing the State Trait Anxiety Inventory, followed by the Davidson Trauma Scale. Next, they answered the Mental Health Continuum Short Form. The other half completed the two groups of questionnaires in the reverse order.

### Measures

#### Anxiety

The State Trait Anxiety Inventory (STAI; [48],validated in Spanish by [49], was used to measure state anxiety. This instrument is composed of two scales, trait anxiety and state anxiety (STAI-S), including 20 items each (e.g., I am tensed). As the objective of the study was to analyze the effect of the COVID-19 pandemic on health-care professionals’ anxiety, we only used the state anxiety scale. Responses to the 20 items were recorded on a 4-point scale ranging from 0 (almost never) to 3 (almost always), therefore total sum scores on the STAI-S can range from 0 to 60, with higher scores indicating higher anxiety levels. The STAI-S has demonstrated good internal consistency, sensitivity to detection of stress, and convergent and discriminant validity in English and Spanish versions [4, 19, 47]. In the present study, Cronbach’s *α* value for the STAI-S scale was 0.93.

#### Trauma intensity

The Spanish version of the Davidson Trauma Scale (DTS; [10],Spanish version: [7] was used linked with COVID-19 pandemic to obtain a general dimensional measure of trauma intensity. The DTS is a 17-item self-report questionnaire of posttraumatic stress symptoms (e.g., Have you had distressing dreams of the event?). Each of the 17 items correspond to the 17 DSM-IV symptoms of Posttraumatic Stress Disorder (PTSD) (items 1–4 and 17 are related to criteria B, intrusive re-experiencing,items 5–11 are linked to criteria C, avoidance and numbness; items 12–16 are related to criteria D, hyperarousal). For each item, participants rated both frequency and severity using 5-point (0–4), Likert-type scales. Total sum scores can range from 0 to 136, with higher scores indicating higher levels of trauma intensity. The DTS demonstrated good internal consistency, factorial, convergent and divergent validity both in the original version and in the Spanish version [13, 15, 35]. In the current study, Cronbach’s *α* for the DTS score was 0.91.

#### Well-being

To measure well-being participants completed the Spanish version of the Mental Health Continuum-Short Form (MHC-SF; [29, 33],Spanish version: [14]. The MHC-SF is a 14-item measure of well-being designed to assess emotional, psychological, and social well-being. Each item represents one theory guided dimension, such as “In the past month, how often did you feel that you had something important to contribute to society?” measuring social contribution of social well-being [24]. This period of one month coincides with the time between the beginning of the first wave in Spain (school closure: March 10th, 2020; national state of emergency and full confinement in Spain: March 14th, 2020) and data collection. Therefore, we thought that the MHC-SF has the potential to capture well-being changes produced by the COVID-19 first wave. The MHC-SF demonstrated excellent internal consistency and factorial validity both in the original and in the Spanish version [14, 29]. Participants responded to items on a 6-point Likert scale ranging from 1 (never) to 6 (every day), therefore total sum scores on the MHC-SF can range from 14 to 84, with higher scores indicating higher levels of well-being. Also, scores of the hedonic-emotional well-being (HWB), psychological well-being (PWB) and social well-being (SoWB) sub-scales were calculated. In the present study, Cronbach’s *α* value for the MHC-SF was 0.90, for the HWB was 0.85, for the PWB was 0.85, and for the SoWB was 0.83.

#### Personal protection equipment availability

Participants answered the question “During the last month, I had access to PPE, in accordance with the COVID-19 technical specifications for health-care professional’s PPE proposed by the government of Spain (Ministry of Health)” using a dichotomous format (Yes/No).

### Data analysis

Pearson correlations were used to examine the relationships between all scales. The level of significance value was set to *α* = 0.05 for all analyses. Also, the confidence intervals of correlation coefficients were computed. In order to test the two continua model of mental health, we analyze whether two different dimensions emerge from the measures used to assess health-care professionals positive health (MHC-SF: emotional/hedonic well-being, psychological well-being and social well-being sub-scales) and illness (PTSD; DTS). Since no prior published studies have examined the positive mental health-mental illness bi-dimensionality in health-care workers, and considering the sample size, we conducted an exploratory factor analysis (EFA) (e.g., [17, 34]. According to our objective, one of the most critical methodological decisions regarding EFA is the number of factors to retain. Following various authors’ recommendations (e.g., [21] to estimate the number of factors to maintain we used parallel analysis (PA). To do so, we used SPSS syntax developed by [39] to calculate the mean for each of the eigenvalues of a 100 randomly generated data sets. The random data eigenvalues were compared to the real-data eigenvalues obtained from a Principal Component Analysis (PCA) in which the number of factors extracted equals the number of scales entered into the analysis. Following the criterion established by the PA of the number of factors to be extracted, an EFA was conducted. We employed principal axis as factor extraction method, as Fabrigar et al. [16] suggest. Also, we used direct oblimin as rotation method because, according to our hypothesis, the emerged dimensions were expected to be correlated. To analyze anxiety, well-being, and traumatic intensity differences between health-care professionals with and without PPE availability we conducted different ANOVAS. Partial eta squared (*η*_*p*_^2^) was calculated as measures of effect size. Values of 0.01, 0.06, and values above 0.14 were considered as small, medium, and large, respectively. Finally, to test our hypothesis regarding the moderating role of PPE availability in the effect of mental illness (i.e., anxiety and trauma intensity) on positive mental health (i.e., well-being), data were subjected to a hierarchical regression analysis. We introduced predictor variables at the first step, then added a computed interaction term at the second step. Gender and Profession were entered as covariables using Process Syntax [20]. Also, confidence intervals were computed.

## Results

Table 1 presents Means, Standard Deviations and Pearson correlation coefficients among DTS, STAI-S, and MHC-SF. In general, health-care professionals showed high levels of anxiety and traumatic intensity. In fact, 80 health-care professionals (34.48%) reported a DTS score greater than 40 which is the optimal cut-point for accurate classification of those with or without PTSD (efficiency = 0.83) according to Davidson and colleagues (1997). Moreover 125 participants (53.88%) had a score greater than 32, a cut-point score with an efficiency of 0.94 according to McDonald and colleagues (2009) to correctly classify the presence or absence of any anxiety disorder. Although there is not a consensus regarding STAI-S cut-off points, we used the criterion proposed by [31] that sets a cut-off point of 35 with an efficiency of 0.87. According to this criterion, sixty-four health-care professionals (70.69%) presented a STAI-S score greater than 35, which could indicate the presence of anxiety disorders. Regarding the presence of positive health, only 52 health-care professionals (22,415) showed a flourishing mental health diagnosis based on Keyes [26] criteria (participants experienced at least 1 of the 3 HWB symptoms and 6 of the 11 PWB/SoWB symptoms ‘every day’ or ‘almost every day’ in the past month).

**Table 1:** Means, standard deviations, Pearson’s correlations and 95% confidence intervals of STAI-S, DTS, and MHC-SF

	Mean	SD	STAI-S	DTS	MHC-SF
STAI-S	40.31	10.39		.67** [.59 .73]	− .38** [− .25 to .50]
DTS	33.15	13.67			− .37** [− .25 to .47]
MHC-SF	53.45	12.31			
HWB	9.97	3.46	− .47** [− .34 to .59]	− .43** [− .31 to .54]	.77** [.70 .83]
PWB	23.21	6.20	− .34** [− .21 to .46]	− .32** [− .20 to .43]	.89** [.86 .92]
SoWB	20.26	5.05	− .18** [− .06 to .30]	− .19** [− .06 to .31]	.78** [.72 .84]

**p < .01

As expected, the two pathology measures were significantly correlated with each other. Moreover, in line with the Complete State Model of Health, both measures of pathology were also negatively correlated with the indicators of presence of positive health (i.e., MHC-SF). Considering the MHC-SF subscales, social well-being shows the weakest correlations with pathology measures.

To test the two-continua model of positive mental health (i.e., MHC-SF) and mental illness (i.e., STAI-S; DTS), we first conducted a PA. Only the first and second eigenvalues of the real dataset (i.e., 2.67, 1.19) exceeded mean random values (i.e., 1.09, 1.07). Five variables were introduced into the EFA analysis to test factor loadings of all MHC-SF subscales, HWB, PWB, SoWB, STAI-S and DTS. The *N*:*p* ratio was 46.4, higher than those generally recommended in the literature to yield factors’ good recovery (e.g., [18], and communalities were relatively high (all greater than 0.55 except for SoWB that was 0.45), indicating a good factor recovery [23]. All the sub-scales of MHC-SF essentially loaded on the first factor (53.43% of variance explained) and the STAI-S and DTS loaded on the second factor (21.86% of variance explained) (Table 2). These results support the two-continua model of mental health: positive mental health (MHC-SF; factor 1) and mental illness (STAI-S and DTS; factor 2). The correlation between factors was − 0.34, which is a first indicator of the relationship between mental illness and positive mental health.

**Table 2:** Exploratory factor analysis of MHC-SF subscales (HWB, PWB, and SoWB), STAI-S and DTS

	1	2
HWB	.76	
PWB	.85	
SoWB	.83	
STAI-S		.91
DTS		.90
%Variance	53.43	75.29
Factors correlation		− .34

Presented is the structure matrix of a principal axis extraction with direct oblimin rotation. The table only presents loadings above .40

Regarding the importance of PPE accessibility for professionals’ mental health, seventy health-care professionals (30.2%) indicated that they had access to the PPE, and 162 (69.8%) indicated that they had not. Participants who had access to PPE reported lower levels of state anxiety and traumatic intensity than those who did not have access to the equipment. Concerning the presence of positive mental health, professionals with access to PPE informed of greater well-being compared to those without access (Table 3).

**Table 3:** Means, standard deviations, and one-way analyses of variance in state anxiety, traumatic intensity and well-being

Measure	With PPE		Without PPE		F (1,227)	η_p_^2^
	M	SD	M	SD		
State anxiety	37.84	10.47	41.35	10.20	3.65*	.02
Traumatic intensity	29.90	13.32	34.54	13.63	4.92*	.02
Well-being	59.16	11.79	51.01	11.73	23.19***	.09

*p < .05

***p < .001

**Fig. 1:**
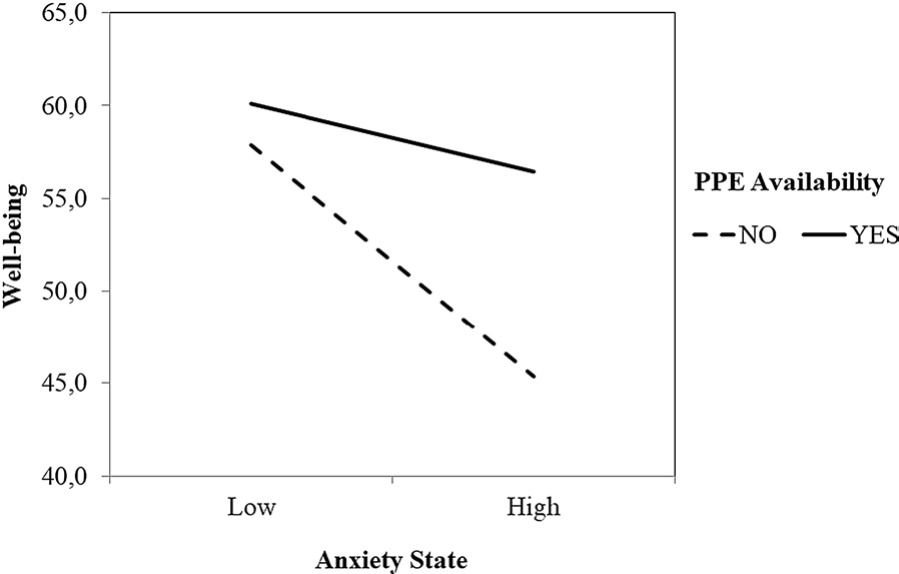
Well-being as a function of Anxiety State and PPE Availability. Note: Well-being was measured using the Mental Health Continuum Short Form. Anxiety State was measured using the State Trait Anxiety Inventory. PPE: Personal Protection Equipment

**Fig. 2:**
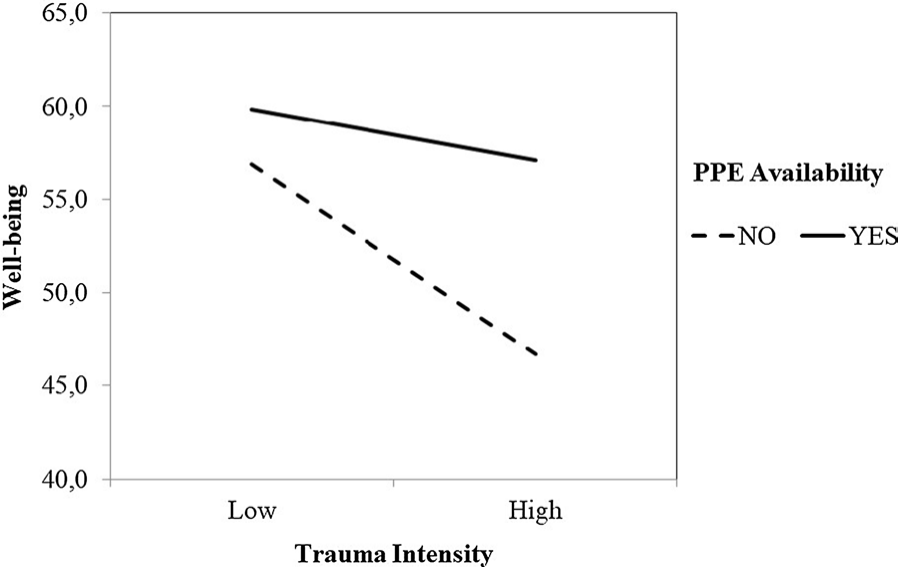
Well-being as a function of Trauma Intensity and PPE Availability. Note: Well-being was measured using the Mental Health Continuum Short Form. Trauma Intensity was measured using the Davison Trauma Scale. PPE: Personal Protection Equipment

Finally, we expected PPE availability to moderate the relationship between mental illness and positive mental health. Specifically, PPE availability should moderate the relationship between anxiety and well-being. To test our hypothesis, MHC-SF was subjected to a hierarchical regression analysis. We introduced PPE availability and STAI-S (centered score) as predictor variables at the first step and added a computed interaction term at the second step. Gender and Profession were entered as covariables and both were no significant, *B* = 0.01, *t* (226) = 0.15, *p* = 0.88, 95% CI [− 0.11, 0.13], *B* = 0.09, *t* (226) = 1.49, *p* = 0.14, 95% CI [− 0.03, 0.20]. As expected, this analysis revealed that the main effect of PPE availability, *B* = −0.56, *t* (226) = −4.34, *p* < 0 0.01, 95% CI [− 0.82, − 0.31] and the main effect of STAI-S, *B* = −0.41, *t* (226) = −6.52, *p* < 0.01, 95% CI [− 0.54, − 0.29] were significant. Most relevant for the purposes of the present analysis, the data revealed a significant PPE availability × STAI-S interaction, *B* = −0.36, *t* (226) = −2.99, *p* < 0.01, 95% CI [− 0.60, − 0.12]. As depicted in Fig. 1, this interaction revealed that among participants without PPE availability, STAI-S were strongly related with MHC-SF, *B* = −0.52, *t* (226) = −6.47, *p* < 0.01, 95% CI [− 0.68, − 0.36]. This relationship was not significant among participants who reported PPE availability, *B* = −0.16, *t* (226) = −1.75, *p* = 0.08, 95% CI [− 0.34, 0.02]. Also, we expected the relationship between trauma intensity and well-being to be moderated by PPE availability. Similarly, MHC-SF was subjected to a hierarchical regression, with PPE availability and DTS as predictor variables. Again, the covariables Gender and Profession were not significant, *B* = 0.06, *t* (226) = 1.00, *p* = 0.32, 95% CI [− 0.06, 0.17], *B* = 0.05, *t* (226) = 0.80, *p* = 0.42, 95% CI [− 0.07, 0.16]. The main effects of PPE availability, *B* = −0.55, *t* (226) = −4.17, *p* < 0.01, 95% CI [− 0.80, − 0.29] and DTS, *B* = −0.32, *t* (226) = −5.44, *p* < 0.01, 95% CI [− 0.44, − 0.21] were significant. According to our hypothesis, the PPE availability × DTS interaction was also significant, *B* = −0.29, *t* (226) = −2.18, *p* = 0.03, 95% CI [−0.55, − 0.03]. This interaction reveled that among participants without PPE availability, DTS was strongly related with MHC-SF, *B* = −0.41, *t* (226) = −5.78, *p* < 0.01, 95% CI [− 0.55, − 0.27]. However, this relationship was not significant for participants who reported having access to PPE, *B* = −0.12, *t* (226) = −1.12, *p* = 0.27, 95% CI [− 0.34, 0.09] (Fig. 2).

## Discussion

According to WHO recommendations, the main objective of the present research was to evaluate health-care professionals’ mental health during COVID-19 first wave using instruments designed not only to measure psychopathology (i.e., anxiety and stress), but also to measure the presence or absence of well-being.

As in previous studies (e.g., [37], our participants showed high levels of anxiety and traumatic intensity. There are different reasons that can explain these results. First, the reorganization in hospitals and the work overload caused by the COVID19 led to job stress [11]. Also, healthcare professionals had a constant fear of COVID-19 disease exposure. Moreover, they were afraid not only to get infected, but also to infect their families, given that the Spanish Government did not provide alternative accommodation to healthcare professionals, not even to those directly involved in the care of patients with COVID-19. Some of them decided to isolate themselves from their family and friends using their own resources, but this choice limited normal adaptive coping strategies, especially social interactions. Finally, death became present in everyday life, both in healthcare contexts and through the mass media, making the inevitability of mortality salient [8], a fact that may also increases anxiety [36, 43].

Regarding positive mental health, participants presented relatively low levels of well-being indicators (i.e., MHC-SF). The COVID-19 pandemic considerably reduced people’s positive emotions and live satisfaction [9, 55], and therefore could have affected health-care professionals HWB. There are also several reasons why participants’ PWB decreased during the COVID-19 first wave. For example, COVID-19 pandemic increased feelings of uncertainty [42]. Also, many professionals attend patients without having the required clinical training. Both factors may lead to less environmental mastery, that is a core dimension of PWB. Moreover, health-care professionals reduced social interactions with their closed ones to protect them from possible contagion [31], which could affect positive relations with others, another important indicator of PWB. Also, reorganization in hospitals and work overload probably reduced autonomy. Regarding SoWB, COVID-19 pandemic first wave resulted in lockdown measures limiting social contact, thus affecting social integration. Finally, COVID-19 first wave clearly affected social actualization, that is the evaluation of the potential and trajectory of a society, a key component of SoWB.

Another important aspect of our study is the relation between illness and positive mental health. Both measures of pathology (i.e., STAI-S and DTS) were negatively related with well-being (i.e., MHC-SF), although the subscale of SoWB showed the weakest relation with both anxiety and traumatic intensity. Given that this scale measures well-being from a macrosocial perspective, this weak relationship is probably due to the social recognition health-care professionals received during the first wave of COVID-19. An example of social recognition and gratitude were daily applauses dedicated to health-care professionals at 8 p.m., a behavior that was later adopted by other European countries. Another reason that could justify this weak relationship is health-care professionals’ perception of being necessary to the community, an essential element of social contribution, one of the fundamental indicators of SoWB according to the Complete State Model of Health (e.g., [24].

Our results also verified the two-dimensional positive mental health—mental illness hypothesis when evaluating health-care professionals’ mental health. As expected, the results of Horn's PA and EFA indicated that the measures of pathology (i.e., STAI-S and DTS) and positive mental health (i.e., MHC-SF) loaded on separate but correlated factors. This result is especially relevant because as positive mental health and mental illness symptoms constitute two unipolar dimensions, to comprehensively assess health-care professionals’ mental health we need to assess both the presence/absence of psychopathology and the presence/absence of well-being. Also, this finding has relevant clinical implications. Psychological treatments and interventions not only should be based on reducing psychopathological symptoms using traditional approaches such as cognitive behavioral therapy or prolonged exposure therapy, but they also need to focus on well-being promotion. Well-being therapy [41] or Written Disclosure Procedure [46] could be good alternative treatments to enhance well-being. 

Finally, our last objective was to analyze the importance of PPE availability for health-care professionals’ mental health. This variable has been widely examined in relation to physical health of professionals directly exposed to COVID-19 (e.g., risk infection reduction and nosocomial transmission; see [30, 38, 51]. However, we expected that PPE availability would also affect professionals’ mental health. The present results confirm our prediction. Professionals who had access to PPE informed of lower levels of state anxiety and traumatic symptoms than those who did not, although, in both cases, the effect sizes were small-medium. In addition, from a positive health perspective, professionals with access to PPE reported greater levels of well-being. Several reasons could explain why PPE availability may be a protective factor for health-care professionals’ mental health. First, professionals who participated in the present study were all working in the front-line of COVID-19. Therefore, the absence of adequate protective equipment could significantly rise the fear of contagion, and consequently increase state anxiety and traumatic intensity of the experience [28]. Furthermore, as commented before, in Spain no alternative accommodation was provided to front-line professionals, so they had to return home after work. Not having adequate protective equipment available would increase the possibility of infecting the family despite isolation measures. Consequently, well-being could be significantly reduced. Finally, one of the most important indicators of social well-being is the ability of a society to integrate and embrace its members [5, 12, 24, 53]. In this sense, a clear indicator that society cares about professionals’ health is by providing the appropriate PPE. Therefore, the unavailability of PPE could decrease social well-being. This fact is especially relevant because only 30.2% of the first-line health-care professionals in our study reported having access to the recommended PPE. Actually, this was a reason of constant protests from health-care professionals during the first wave [38].

Since PPE availability is crucial for health-care professionals’ mental health, we also expected that it would moderate the effects of state anxiety and traumatic intensity on well-being. Previous studies showed that anxiety and trauma affect well-being [3, 6, 13] Schnurr et al. (2006), and that perceived control could moderate this relation [50, 54]. In this sense, probably access to PPE increased health-care professionals perceived control, thus reducing the impact of anxiety and trauma on well-being. The analyses carried out confirmed both moderations, being the effect of psychopathological symptoms on well-being lower when participants had access to PPE than when they did not.

## Conclusions

As in previous research, COVID-19 first wave affected health-care professionals in terms of more psychopathological symptoms (i.e., anxiety and traumatic intensity). The novelty of the present research is twofold. First, our results showed that the pandemic reduced health professionals’ well-being. In fact, professionals’ well-being and mental illness reflect distinct continua of mental health, rather than the extreme ends of a single spectrum. Therefore, as a clinical application of our findings, it is essential to measure both psychopathology and the presence of positive health to comprehensively evaluate professionals’ mental health. Importantly, future research should be focused on developing positive psychological treatments to improve well-being of this specific population. Second, our results showed that access to PPE is essential for professionals’ both physical and mental health. Consecutively, governments should guarantee PPE availability as a strategic priority.

## Data Availability

The datasets used and/or analyzed during the current study are available from the corresponding author on reasonable request.

## Abbreviations

CI: Confidence interval
COVID-19: Coronavirus disease 2019
ECDC: European centre for disease prevention and control
DSM: Diagnostic and statistical manual of mental disorders
DTS: Davidson trauma scale
EFA: Exploratory factor analysis
HWB: Hedonic well-being
ICU: Intensive care units
MHC-SF: Mental health continuum short form
PA: Parallel analysis
PPE: Personal protection equipment
PTSD: Posttraumatic stress disorder
PWB: Psychological well-being
SoWB: Social well-being
STAI: State TRAIT ANXIETY INVENTORY
WHO: World health organization
